# Psychological well-being during the COVID-19 pandemic in Italy assessed in a four-waves survey

**DOI:** 10.1038/s41598-022-22994-4

**Published:** 2022-10-26

**Authors:** Giovanni de Girolamo, Clarissa Ferrari, Valentina Candini, Chiara Buizza, Gemma Calamandrei, Marta Caserotti, Teresa Gavaruzzi, Paolo Girardi, Katrine Bach Habersaat, Lorella Lotto, Martha Scherzer, Fabrizio Starace, Alessandra Tasso, Manuel Zamparini, Cristina Zarbo

**Affiliations:** 1 Psychiatric Epidemiology and Evaluation Unit, IRCCS Istituto Centro San Giovanni Di Dio Fatebenefratelli, Via Pilastroni 4, 25125 Brescia, Italy; 2 Service of Statistics, IRCCS Istituto Centro San Giovanni Di Dio Fatebenefratelli, Brescia, Italy; 3 Centre for Behavioral Science and Mental Health, Istituto Superiore Di Sanità, Roma, Italy; 4 Department of Developmental Psychology and Socialization, University of Padova, Padova, Italy; 5 Department of Statistical Sciences, University of Padova, Padova, Italy; 6 World Health Organization, Regional Office for Europe, Copenhagen, Denmark; 7 Department of Mental Health and Drug Abuse, AUSL Modena, Modena, Italy; 8 Department of Humanities, University of Ferrara, Ferrara, Italy

**Keywords:** Psychology, Human behaviour

## Abstract

COVID-19 pandemic had a negative impact on the mental health and well-being (WB) of citizens. This cross-sectional study included 4 waves of data collection aimed at identifying profiles of individuals with different levels of WB. The study included a representative stratified sample of 10,013 respondents in Italy. The *WHO 5-item well-being scale* (WHO-5) was used for the assessment of WB. Different supervised machine learning approaches (multinomial logistic regression, partial least-square discriminant analysis—PLS-DA—, classification tree—CT—) were applied to identify individual characteristics with different WB scores, first in waves 1–2 and, subsequently, in waves 3 and 4. Forty-one percent of participants reported “Good WB”, 30% “Poor WB”, and 28% “Depression”. Findings carried out using multinomial logistic regression show that R*esilience* was the most important variable able for discriminating the WB across all waves. Through the PLS-DA, *Increased Unhealthy Behaviours* proved to be the more important feature in the first two waves, while *Financial Situation* gained most relevance in the last two. *COVID-19 Perceived Risk* was relevant, but less than the other variables, across all waves. Interestingly, using the CT we were able to establish a cut-off for *Resilience* (equal to 4.5) that discriminated good WB with a probability of 65% in wave 4. Concluding, we found that COVID-19 had negative implications for WB. Governments should support evidence-based strategies considering factors that influence WB (i.e., *Resilience, Perceived Risk, Healthy Behaviours, and Financial Situation*).

## Introduction

The ongoing COVID-19 pandemic has produced negative health consequences beyond those caused by the virus per se*,* as shown by several cross-sectional population studies^1^. Longitudinal studies conducted during the COVID-19 pandemic provided more accurate data about the dynamics of changes in mental health indicators caused by the emotional response to the pandemic, lockdown and other profound alterations of ordinary life conditions that had to be adopted to limit the spread of infection^2,3^, with disruptions of social ties and work habits^4^. Psychological distress due to the pandemic was increased by the reduction in social contacts, forced isolation, and quarantine^5^. Recent studies have identified several individual characteristics that represent risk factors for mental health during the COVID-19 outbreak, and they include young age, female gender, being a blue-collar worker employed in ‘essential’ jobs, low income, being unemployed, having a preexisting mental health condition, and physical inactivity^4,6–9^.

The pandemic severely affected Italy, a country with over 60 million inhabitants, and as of 6th October, 2021, 4,691,036^10^ COVID-19 cases have been reported and 130,508 deaths have been officially attributed to SARS-COV-2 disease. Interestingly, a large study^11^ conducted in April-June 2020 on 9565 people from 78 countries and 18 languages found that Italians reported the lowest levels of well-being (WB). Several cross-sectional studies have been conducted in Italy to estimate the magnitude and characteristics of ill-health among the general population^12,13^, but very few focused on WB status. Moreover, almost all Italian surveys were conducted on convenience samples obtained through ad-hoc recruitment via social media or self-administered questionnaires spread via mailing lists, Facebook, newspapers, etc. Very few studies have applied detailed sampling strategies, with repeated waves to estimate changes in self-reported WB in parallel with significant changes in the dynamics of the pandemic (e.g., rate of contagion, number of hospitalizations, etc.), with the introduction or dismissal of restrictive measures and finally with the availability of key preventive measures, such as vaccines.

In this context, to grasp a more fine-grained picture of the overall psychosocial impact of the pandemic on the general adult population, the WHO Regional Office for Europe promoted a large survey entitled “*Monitoring knowledge, risk perceptions, preventive behaviours and trust to inform pandemic outbreak response”* to which more than 30 countries and areas in this Region, including Italy, joined. Approximately 1 year after the start of the pandemic, we conducted a 4-wave survey in a large, stratified Italian sample involving 10,013 citizens; the survey was conducted in January, February, March and May 2021.

The aims of this paper were threefold: (a) to investigate the sociodemographic, psychological, and individual differences of people with different degrees of psychological self-reported WB, as assessed by the WHO-5 questionnaire (i.e., Good WB; Poor WB; Depression); (b) to identify profiles of individuals with different degrees of psychological WB during the first 2 waves (considered to be homogeneous in terms of restrictive measures, January–February 2021); and (c) to explore whether the WB profiles identified in the first 2 waves changed in the subsequent 2 waves, when restrictive measures were gradually phased out and vaccines became available to a significant proportion of the adult population (March and May 2021).

## Methods

### Participants and procedures

The Italian survey “*COVID Monitoring in Italy* (“COMIT”, see also^14,15^; Registered ISRCTN on 11/05/2021, ID: ISRCTN26200758) was conducted in four waves (January-May 2021) through an online questionnaire developed ad hoc by WHO Regional Office for Europe and the University of Erfurt and administered to a sample of 10,013 individuals aged 18–70 years. The online survey was restricted to people younger than 70 years-old due to a possible selection bias on access to the internet and/or ability to complete the web questionnaire. On the other hand, we exclude individuals aged younger than 18 years for ethical considerations related to gaining consent from minors. Each wave included approximately 2500 participants (1st wave: 2504 individuals, 2nd wave: 2502 individuals, 3rd wave: 2507 individuals, 4th wave: 2500 individuals.). Each wave surveyed different participants, hence these were 4 different cross-sectional surveys involving different representative general population samples. See supplementary materials for a deep understanding about the state of the pandemic in Italy during the period in which the study was conducted.

The field survey was conducted by Doxa S.p.A. and carried out with the computer assisted web interviewing technique (CAWI) on an online panel and on the Confirmit software platform used by Doxa S.p.A. A detailed sampling plan was developed to obtain a representative stratification of the Italian adult population. The following variables were taken into account to stratify the participants: gender by age (18–34 years, 35–44 years, 45–54 years, 55–70 years); geographical area (Northwest, Northeast, Centre, South and Islands); size of living centers (above and below 100,000 inhabitants); education level (up to lower middle school, beyond lower middle school); and employment (employed, not employed). All participants received an invitation by e-mail to fill the online interview via a link: first, informed consent was requested and then the questionnaire was accessed. Participants freely decided to participate to the study, with no financial incentive. The average administration time was approximately 18–20 min.

At the end of each wave, a weighting procedure was applied to accurately restore the proportionality of the total sample examined with the reference population, according to the most recent data of the Italian Statistics Institute (ISTAT, 12/31/2019). In particular, data were weighted for the main sociodemographic and geographic variables (e.g., sex by age by geographical area, occupation, education, geographical area by the size of living cities/towns). The reported sample size was chosen to maintain a sampling error of less than 2% (at the significance level of 95%) and to control the error of the estimates within the groups or subgroups of interest.

### Measures

The WHO Regional Office for Europe questionnaire includes 21 different thematic areas noteworthy for the investigation of the COVID-19 experience. The questionnaire was translated into specific country languages by each recruiting site, following the WHO’s standard procedures for translations of tools into other languages. The process included the following steps: forward translation, panel experts, back-translation, pretest and cognitive interviews, and, finally, development of the final version. In this paper we considered the following domains of the WHO questionnaire: sociodemographic characteristics (i.e., age, sex education level, occupational status, Italian region, chronic illness, cohabiting, and financial situation); *Financial Concerns; COVID-19 Experience* (i.e. having been infected or knowing someone infected); *COVID-19 Perceived Risk*, including items regarding the perception of the Probability, Vulnerability, and Severity of getting the virus and two items about the related-affect (i.e. Affect-Frightening and Affect-Closeness); *Beliefs About Vaccine Efficacy* (i.e. beliefs about vaccine efficacy in reducing the spread of COVID-19); *Trust in Healthcare Institutions* (i.e., family doctor, hospitals, Ministry of Health, National Health Institute); *Increased Unhealthy Behaviours* (i.e., changes of physical activity, diet, smoking, drinking alcohol, medical seeking) during the previous 2 weeks; and *Resilience*, through three items of the five that compose the Brief Resilience Scale^16^.

The WHO 5-item well-being scale (WHO-5)^17^ was used for the assessment of WB. The WHO-5 is a widely used measure of well-being comprised of five items ranging from 0 (at no time) to 5 (at all times), which indicates the subjective quality of life based on positive mood (good spirits, relaxation), vitality (being active and waking up fresh and rested), and general interest (being interested in things)^17^ in the previous 2 weeks. The score is calculated by summing the score of each item^17^, and the raw score ranges from 0 (absence of WB) to 25 (maximal WB). Because scales measuring health-related quality of life are conventionally translated to a percentage scale from 0 (absent) to 100 (maximal), it is recommended to multiply the raw score by 4. A score of 51–100 indicates good WB (“Good WB” group), and a score ≤ 50 indicates poor psychological WB (“Poor WB” group) which may suggest further investigation for possible symptoms of depression^18^. A score less than or equal to 28 may be indicative of clinical depression (“Depression” group)^19^.

See table 6S for a detailed list of instruments and their domains.

### Statistical analyses

Descriptive statistics were carried out through means and standard deviation (SD) for continuous variables or frequencies and percentages for categorical variables. The Kolmogorov–Smirnov and Shapiro–Wilk tests were used to assess whether continuous variables were normally distributed. The Chi-square test was applied to compare WB groups distribution across waves. Group comparisons in terms of mean scores were performed with ANOVA or corresponding nonparametric Kruskal–Wallis test according to the distribution of the scores (Gaussian and non-Gaussian respectively). Differences of categorical variables distribution across groups were tested by Chi-Square tests.

Given the large number of items of the WHO questionnaire, a data reduction procedure based on factor analysis was applied to derive some factors that summarize prominent domains related to WB. More specifically, the variables *COVID-19 Perceived Risk* and *Trust in Healthcare Institutions* were obtained by applying an exploratory factor analysis on the following items: Probability, Vulnerability, Severity, Affect-Frightening and Affect-Closeness and Trust in Family Doctors, Hospitals, Ministry of Health, and Italian National Health Institute (see the details in Table 5S).

To identify profiles of individuals with different degrees of psychological WB, three different supervised classification approaches (based on a machine learning strategy) were applied. An initial analysis was conducted to assess the association between the WB groups and the participants’ features: multinomial (univariate) logistic regression models were applied on WB groups (as a dependent variable) and sociodemographic, clinical and individual variables as independent variables. The relevant features associated with WB groups were assessed in terms of significance (*p*-value) and in terms of the strength of the association (expressed in terms of Nagelkerke’s R^2^ [N-R^2^]). Subsequently, a variable selection procedure, through the Partial Least Squares Discriminant Analysis (PLS-DA)^20^, was carried out to define which variables mainly contributed to discriminate among the WB groups. This multiple supervised approach (with WB groups as dependent variables) was performed to detect any possible confounding effects among the sociodemographic, clinical and individual variables: the coherence between logistic models and the PLS-DA ensured robust outputs in both univariate and multiple frameworks. Finally, a Classification Tree (CT) was carried out (on the WB groups, as a categorical dependent variable to be predicted), to identify different classification pathways defined by estimated covariate cut-offs. The CT was carried out on a training set of the 80% and a test set of 20% of the whole sample. A tree pruning strategy (based on complexity parameter of the *tree* function in *R-*package *tree*) was adopted to reduce CT overfitting^21,22^. In addition, accuracy indices were computed to evaluate the classification performances of the CT considering the WB outcome in three and in two (Depression vs Good WB) categories. A comparison of the results obtained by logistic models and PLS-DA were used to confirm and validate the CT results. The three methods address the classification problem in slightly different ways, and they are based on different assumptions and computations. The choice to perform the three methods and to compare their results was aimed at ensuring robustness of findings. The multinomial logistic model estimates the parameters maximizing the likelihood of the observed data (e.g. in the method called Maximum Likelihood Estimator—MLE); the PLS-DA and CT are two non-parametric methods aiming to i) provide a dimension reduction in a discriminant application maximizing among-groups variability, and to ii) classify following a series of logical if–then conditions (tree nodes), respectively. Methodological details on the machine learning approaches are provided in the Supplementary Materials.

These three methods were first applied to waves 1–2 (which were considered homogeneous in terms of pandemic spread and restrictive measures) and subsequently to wave 3 and wave 4 (separately) to explore any changes in the identified WB profiles over waves sampled at different times.

## Results

### Sociodemographic and clinical characteristics of the overall sample

**Figure 1:**
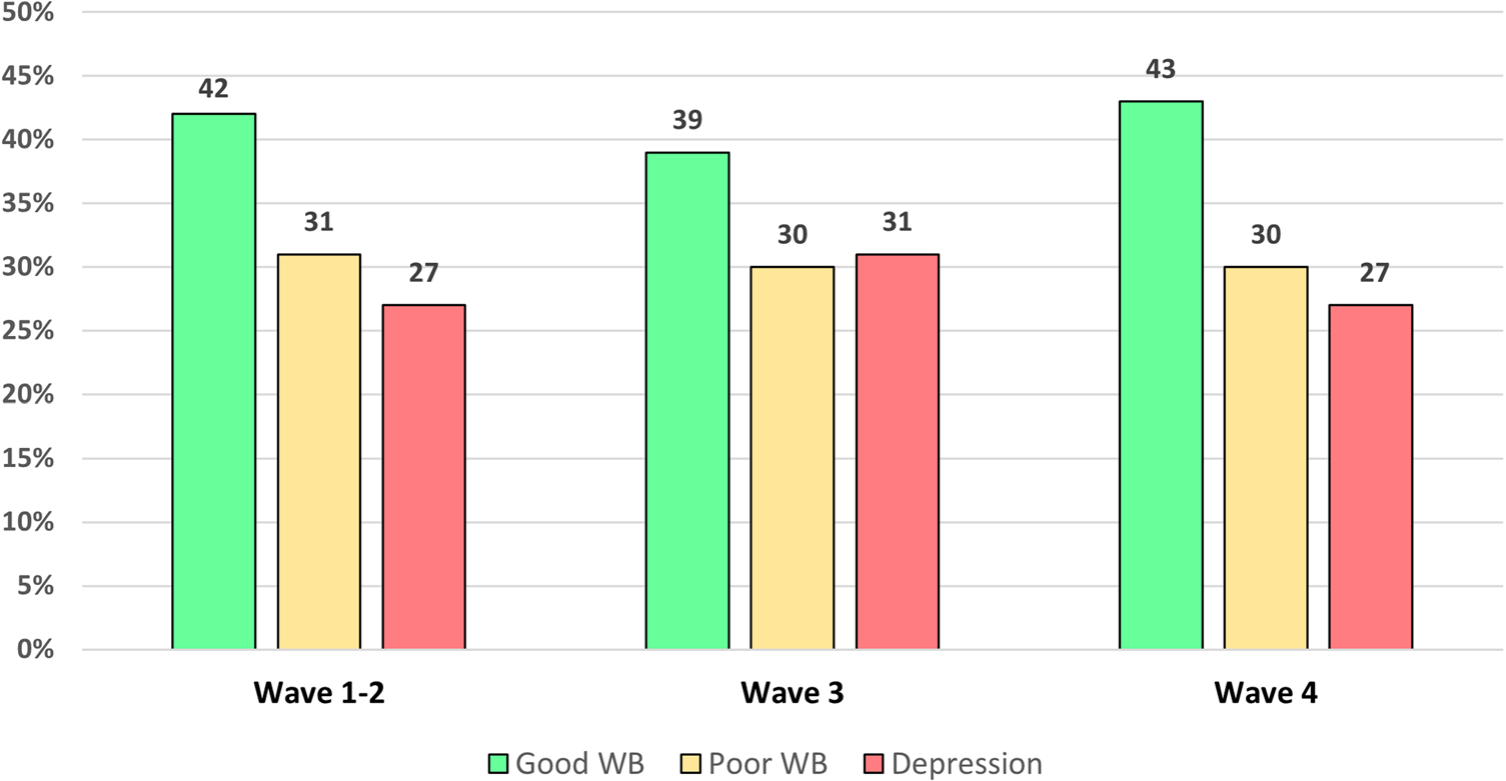
Percentages of WHO-5 categories across 4 waves in the Italian survey (N = 10,013).

The overall mean age of the four waves final sample was 45.6 years (SD = 12.8 years) and 49.6% were men. Among the 10,013 assessed participants, 41.4% showed “Good WB”, 30.5% “Poor WB”, and 28.1% “Depression”. In the first and second waves, 42.0% of the sample showed “Good WB”, 31.1% “Poor WB”, and 26.9% “Depression”. This distribution did not significantly change over the waves (Fig. 1, Fig. 1S). WHO-5 mean scores across the four waves were: 11.36 (SD = 5.54) (1st wave), 11.33 (SD = 5.56) (2nd wave), 10.86 (SD = 5.70) (3rd wave), 11.43 (SD = 5.55) (4th wave). The mean WHO-5 score of the four waves was 11.25 (SD = 5.59).

The baseline characteristics of the overall sample (N = 10,013, equally distributed across the 4 waves) are shown in Table 1S. Almost all variables regarding sociodemographic features, *COVID-19 Perceived Risk*, *COVID-19 Experience*, *Trust in Healthcare Institutions* and other behavioural characteristics were differently distributed across the WB groups (*p* < 0.001, for almost all variables).

Based on preliminary analyses and on the available literature, we identified 15 sociodemographic, clinical and individual variables potentially associated with different degrees of self-reported WB: among these variables, only 11 were associated with a statistically significant difference between WHO-5 groups in the first two waves (Table 1). In terms of sociodemographic features, groups reporting “Good WB” were mainly men (56.8%) and were slightly older (Age mean = 46.7, SD = 12.8) than individuals in the “Depression” or “Poor WB” groups (means 46 and 45, SD = 12.5 and 12.9 respectively) (*p* < 0.001). Significant differences between WHO-5 groups were also found for *Education*, *Occupational Status* and *Financial Situation*, while no differences were evident in terms of geographical regions. Interestingly, personal experience with COVID-19 was not associated with the WB groups which, instead, were related to *Increased Unhealthy Behaviours*, *Beliefs About Vaccine Efficacy* and the *Resilience*.

**Table 1:** Sample composition and selected characteristics according to WHO-5 scores (Wave 1–2).

		Good WB [group 2] N = 2101 (42.0%)	Poor WB [group 1] N = 1560 (31.1%)	Depression [group 0] N = 1345 (26.9%)	Test	*p *value	Bonferroni Post-Hoc test	Effect size
	**SOCIODEMOGRAPHIC INFORMATION**							
*1*	*Age (mean, SD)*	46.67 (12.80)	44.95 (12.93)	45.96 (12.46)	K-W	** < 0.001**	1 < 2	.003
*2*	*Sex*				Χ^2^	** < 0.001**	£ $ &	.146
	Male	1194 (56.8%)	770 (49.4%)	522 (38.8%)				
	Female	907 (43.2%)	790 (50.6%)	823 (61.2%)				
*3*	*Education level (years)*				Χ^2^	**0.007**	£ $	.045
	0–8 years	843 (40.1%)	610 (39.1%)	599 (44.5%)				
	> 8 years	1258 (59.9%)	950 (60.9%)	746 (55.5%)				
*4*	*Occupational status*				Χ^2^	**0.027**	£ $ &	.038
	Yes	1139 (54.2%)	829 (53.1%)	667 (49.6%)				
	No	962 (45.8%)	731 (46.9%)	678 (50.4%)				
*5*	*Health workers*				Χ^2^	0.078	/	.044
	Yes	76 (6.7%)	78 (9.4%)	50 (7.5%)				
	No	1063 (93.3%)	751 (90.6%)	617 (92.5%)				
*6*	*Italian Region*				Χ^2^	0.218	/	.034
	North	1000 (47.6%)	713 (45.7%)	591 (43.9%)				
	Center	394 (18.8%)	292 (18.7%)	280 (20.8%)				
	South & Islands	707 (33.7%)	555 (35.6%)	474 (35.2%)				
*7*	*Chronic Illness*				Χ^2^	** < 0.001**	£ $ &	.088
	Yes	380 (18.5%)	326 (21.7%)	352 (27.6%)				
	No	1669 (81.5%)	1178 (78.3%)	924 (72.4%)				
*8*	Cohabiting*				Χ^2^		/	
	Alone (yes vs no)	215 (10.2%)	147 (9.4%)	150 (11.2%)		0.308		.022
	With other adults (yes vs no)	1695 (80.7%)	1258 (80.6%)	1088 (80.9%)		0.983		.007
	With under 18 (yes vs no)	642 (30.6%)	480 (30.8%)	362 (26.9%)		**0.037**		.036
*9*	*Financial Situation (over the past 3 months)*				Χ^2^	** < 0.001**	£ $ &	.180
	Improved	107 (52.5%)	63 (30.9%)	34 (16.7%)				
	Remains the same	1432 (47.6%)	898 (29.9%)	677 (22.5%)				
	Worse							
		543 (31.1%)	581 (33.3%)	623 (35.7%)				
	**COVID-19 EXPERIENCE**							
*10*	*COVID-19 personal infection*				Χ^2^	0.235	/	.026
	Yes	132 (6.8%)	102 (7.3%)	101 (8.5%)				
	No	1808 (93.2%)	1303 (92.7%)	1094 (91.5%)				
	**COVID-19 PERCEIVED RISK**							
*11*	*COVID-19 Perceived Risk*^#^	− 0.15 (0.82)	0.05 (0.73)	0.18 (0.82)	K-W	** < 0.001**	0 < 1 < 2	.029
	**TRUST IN HEALTHCARE INSTITUTIONS**							
*12*	*Trust in Healthcare Institutions***#**	0.07 (0.99)	0.01 (0.90)	− 0.12 (0.98)	K-W	** < 0.001**	0 < 1/2	.006
	**OTHER AREAS**							
*13*	*Resilience (mean, SD)*	4.53 (1.11)	3.91 (0.98)	3.41 (1.14)	K-W	** < 0.001**	0 < 1 < 2	.155
*14*	*Increased Unhealthy behaviours*				Χ^2^	** < 0.001**	£ $ &	.169
	No	1405 (66.9%)	846 (54.2%)	633 (47.1%)				
	Yes	696 (33.1%)	714 (45.8%)	712 (52.9%)				
*15*	*Belief About Vaccine Efficacy in reducing spread of COVID-19*				Χ^2^	**0.003**	£ $ &	.053
	Yes	1609 (41.5%)	1218 (31.4%)	1048 (27.0%)				
	No	191 (49.4%)	92 (23.8%)	104 (26.9%)				

K-W: Kruskal–Wallis test; X^2^: Chi-square test.

^#^ Variables resulted by application of the Factor Analysis and obtained as the extraction of the first factor applied to single items of the WHO questionnaire. Factor analysis for COVID-19 Perceived risk included the following items: Probability, Vulnerability, Severity, Affect-Frightening and Affect-Closeness. Factor analysis for Trust in Healthcare institutions included the items: Family doctor, Hospitals, Ministry of Health, Institute of Public Health.

£: Good WB versus Poor WB; $ Good WB versus Dep; &: Poor WB versus Dep.

Significant values are in bold.

### Psychological well-being in the first two waves

Although the results of the multinomial logistic regression models showed almost all significant associations of the socio-demographic and risk perception variables with the three WB groups; these features showed only moderate performance (Nagelkerke’s N-R^2^ lower than 0.2) in discriminating among the three WB groups (basically due to the difficulty in discriminating between the “Poor WB” vs the “Depression” group,). *Resilience* (*p* < 0.001; N-R^2^ = 0.177) was the most important variable (among the ones investigated) in explaining the subjects’ WB status (see Table 2), followed by *Increased Unhealthy Behaviours* (*p* < 0.001; N-R^2^ = 0.038,) and *Financial Situation* (*p* < 0.001; N-R^2^ = 0.036).

**Table 2:** Multinomial Logistic regression models output: ranking (based on Nagelkerke’s R^2^) of the socio-demographic and risk perception variables in discriminating the three well-being groups.

Variable	Waves 1–2		Wave 3		Wave 4	
	*p* value	*Nagelkerke’s R*^2^	*p* value	*Nagelkerke’s R*^2^	*p* value	*Nagelkerke’s R*^2^
Resilience	** < 0.001**	0.177	** < 0.001**	0.191	** < 0.001**	0.143
Increased Unhealthy Behaviours	** < 0.001**	0.038	** < 0.001**	0.033	** < 0.001**	0.020
COVID-19 Perceived Risk	** < 0.001**	0.035	** < 0.001**	0.029	** < 0.001**	0.027
Financial Situation	** < 0.001**	0.036	** < 0.001**	0.039	** < 0.001**	0.036
Sex	** < 0.001**	0.024	** < 0.001**	0.025	** < 0.001**	0.007
Chronic Illness	** < 0.001**	0.009	** < 0.001**	0.017	** < 0.001**	0.013
Trust in Healthcare Institutions	** < 0.001**	0.007	**0.013**	0.004	** < 0.001**	0.012
Occupational status	**0.027**	0.002	** < 0.001**	0.010	**0.028**	0.003
Age	** < 0.001**	0.004	**0.004**	0.005	** < 0.001**	0.013
Educational level	**0.007**	0.002	**0.028**	0.003	**0.002**	0.006
Beliefs on vaccine efficacy	**0.002**	0.003	** < .001**	0.009	0.087	0.004

Significant values are in bold.

**Figure 2:**
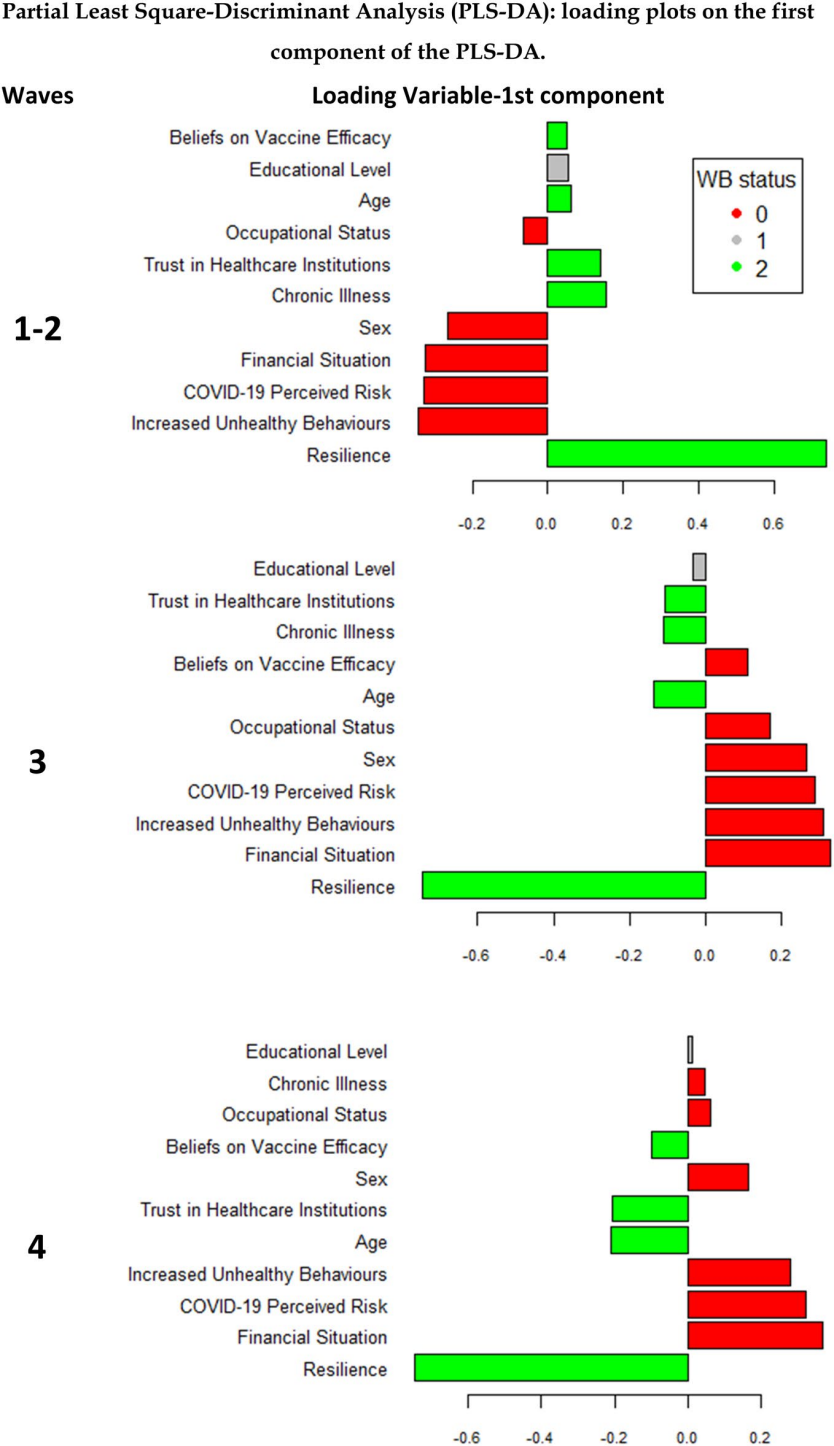
Partial Least Square-Discriminant Analysis (PLS-DA): loading plots on the first component of the PLS-DA. WB status: Well-being status: red 0 = Depression; gray 1 = Poor Well-being; green 2 = Well-being. Loadings plot assigns to each variable bar the sample group colour for which the mean is maximum.

Application of the partial least squares-discriminant analysis technique confirmed *Resilience* and *Increased Unhealthy Behaviours* as the most prominent variables in discriminating between WB groups in a multiple data-reduction approach. In detail, these two variables gave the first and second highest contributions (as defined by the loadings, see Fig. 2 Panel A) in discriminating the “Good WB” and “Depression” status respectively. The “Poor WB” status, an intermediate status, appeared to be the most difficult group to discriminate. With respect to Fig. 2, it is worth noting that the loadings scores should be interpreted considering their absolute values: large loading indicates high discriminant power of the variable in identifying a specific category independently from the sign of the loading and the direction of the variable.

Profiles of individuals with different degrees of psychological WB during the first 2 waves, carried out through classification trees. Overall accuracy of the CT applied to the WB variable was 49%. This value increased to 72% when the CT was applied to WB variable with only two categories (“Good WB” vs “Depression”). Although the accuracy for the three categories WB was quite low (due to the difficulty in the discrimination of the intermediate category “Poor WB”), consistent with previous analyses, we decided to describe the results of the CT for the three categories WB variable. Profiles of individuals were well characterized by *Resilience* and *Trust in Healthcare Institutions.* The consistency of these results with the ones found with the previous analyses confirms their robustness. In particular, individuals who reported *Resilience* scores lower than 2.8 had a 58% chance of belonging to the “Depression” group, and a likelihood of 28% and 15% to belong to the “Poor WB” and the “Good WB” groups, respectively. Conversely, those who had a *Resilience* score higher than 3.8 had a 54% chance of belonging to the “Good WB” group versus 29% and 18% to belong to “Poor WB” and “Depression” groups, respectively. The largest likelihood (41%) of being classified in the “Poor WB” group was reached by respondents who had *Resilience* scores between 3.2 and 3.8, had low scores in *Trust in Healthcare Institutions* and high *COVID-19 Perceived Risk*.

In summary, in the first two waves, the importance of *Resilience* and *Increased Unhealthy Behaviours* emerged as identifier of the profile of “Good WB” group (characterized by high Resilience score) versus the “Depression” group (characterized by increased unhealthy behaviours). The role of *Trust in Healthcare Institutions* was relevant only to identify the “Poor WB” group.

### Psychological WB across time: waves 3 and 4

In the wave 3, *Resilience* and *Financial Situation* were the most important variables in explaining respondents’ WB status in both multinomial logistic regression (Table 2) and PLS-DA analysis: *Resilience* still highly contributed to the discrimination of “Good WB” status, while *COVID-19 Perceived Risk*, *Increased Unhealthy Behaviours* and *Financial Situation* became more relevant in the discrimination of “Depression” status. Notably, variables that seemed to make a substantial contribution to predicting the “Poor WB” status were *Resilience*, *Financial Situation, COVID-19 Perceived Risk* and *Increased Unhealthy Behaviours* (Fig. 2).

**Figure 3:**
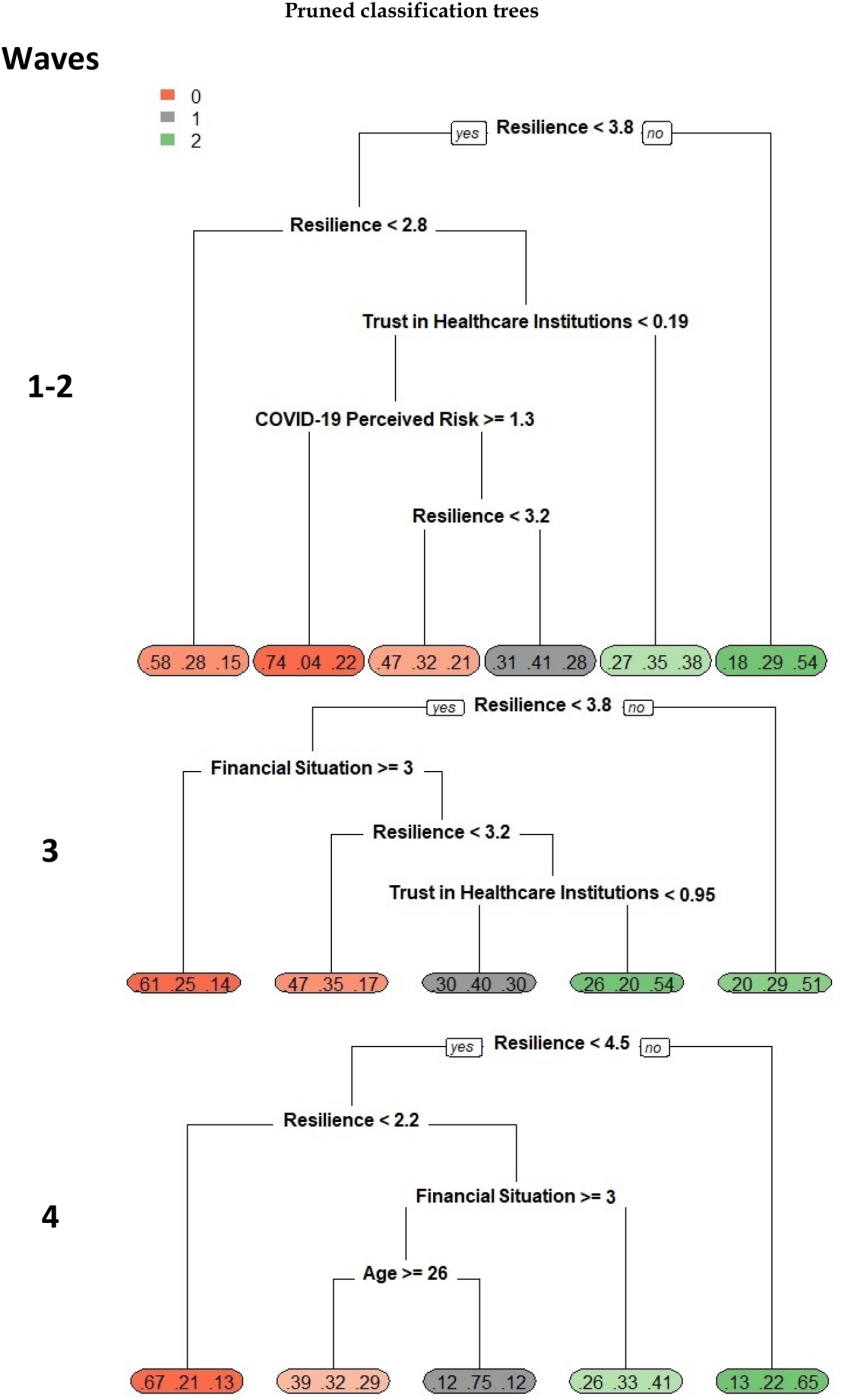
Pruned classification trees. WB status: Well-being status: red 0 = Depression; gray 1 = Poor Well-being; green 2 = Well-being. Resilience range = 1 to 7; COVID-19 Perceived Risk range = -3.3 to 2.5; Trust in Healthcare Institutions range = -2.9 to 1.8; Financial Situation: 1 = Improved, 2 = Remains the same, 3 = Worse.

In line with these results, the classification tree (accuracy = 49% for CT applied to three categories WB, and accuracy = 67% for CT applied to two categories WB) provided profiles of individuals with different degrees of psychological WB well characterized by *Resilience, Financial Situation* and *Trust in Healthcare Institutions* (Fig. 3). Sixty-one percent of participants with a *Resilience* score lower than 3.8 and a worsened *Financial Situation* were classified as having a “Depression” status, while 51% of respondents with a *Resilience* score higher than 3.8 were classified in the “Good WB” group. Compared to the first two waves, in the third wave, a lower *COVID-19 Perceived Risk* contributed to characterizing people with a higher psychological WB.

Finally, in wave 4, multinomial logistic models and PLS-DA both highlighted *Resilience, Financial Situation*, *COVID-19 Perceived Risk* and *Increase Unhealthy Behaviours* as variables contributing to discriminating between “Depression” and “Good WB” status respectively. Interestingly, a worse *Financial Situation* became, over the four waves, the most important variable to discriminate “Depression” status (Table 2 and Fig. 3). Through the classification tree (accuracy = 49% for CT applied to three categories WB, and accuracy = 72% for CT applied to two categories WB) (Fig. 3) the profile of “Good WB” was almost completely identified through *Resilience*: individuals with a score higher than 4.5 had a high probability (65%) of belonging to the “Good WB” group, while respondents with *Resilience* scores lower than 2.2 were more likely to belong to the “Depression” group (67%). Interestingly, in case of a worsened *Financial Situation, Age* played a role in classifying the “Poor WB” group among participants who had intermediate *Resilience* scores (between 2.2 and 4.5).

*Financial Situation* gradually gained importance over time, and *Resilience* was confirmed as an important feature to discriminate the WB status across all waves. Conversely, while the role of *Increased Unhealthy Behaviours* in the first two waves was relevant, it became weaker in the third wave and negligible in the last wave. Interestingly, there was a substantial overlap of the two groups “Depression” and “Poor WB” over time, with a similar likelihood of inclusion found for these two groups in wave 4.

## Discussion

This study aimed to identify socio-demographic, psychological, and behavioural characteristics that discriminate different levels of WB among the general Italian population aged 18–70 years during four different time periods during the COVID-19 pandemic. The large size of the dataset allowed for  addressing the study aims by applying different machine learning techniques in a very efficient way, producing robust and consistent results across all analyses. The application of the logistic multinomial model, PLS-DA and of the classification tree allowed for analyzing the predictors of WB under different and exhaustive perspective. Through the univariate multinomial logistic regression models, we identified the best predictors (in terms of explained variability valuated by N-R2) of the WB. The PLS-DA confirmed the former results in a multivariate context by providing a rank of the variables able to well discriminate WB categories. Finally, through the application of the CTs, the identification of the directionality of the predictors in classify the WB categories has been provided by the cut-offs. Consistentlyacross the methods, our findings show that *Resilience, Financial Situation, COVID-19 Perceived Risk* and *Increased Unhealthy Behaviours* were relevant to discriminate WB levels across different periods. In particular, *Resilience* was confirmed as a key factor across the four waves, with high scores associated with “Good WB”. *Increased Unhealthy Behaviours* was more important in the first two waves, while *Financial Situation* gained most relevance in the last two. *COVID-19 Perceived Risk* was important, but less than the other variables, across all waves.

### WB of the Italian population during the COVID-19 pandemic

Our study shows that the COVID-19 pandemic was associated with widespread self-reported psychological ill-health, with only 41% of all surveyed participants reporting “Good WB”, opposed to 59% showing different degrees of psychological difficulties (i.e., 30% “Poor WB”, and 28% “Depression”). Other studies have investigated WB with WHO-5 in general population samples during the pandemic. As shown in Table 5S, the proportion of respondents reporting “Good WB” during COVID-19 pandemic varies widely across different surveys, ranging from 8.7^23^ to 85.8%^24^; on average 52.6% of respondents report “Good WB”. Only one other study has used multiple waves^25^, and only two studies^24,25^ have been conducted in Italy. Furthermore, almost all studies have been conducted in the first 6 months after the pandemic onset (i.e., until September 2020), and they used different cut-offs and categorization methodologies to classify WB levels. Even considering the limitations of these studies, the percentage of respondents with “Good WB” found in our study (i.e., 41%) was lower than the mean percentage of “Good WB” weighted by the number of participants found by other studies (shown in Table 5S) conducted in 2020 (i.e., 52.6%) during the initial and acute phases of the pandemic. This may indicate an even greater psychological burden associated with the pandemic 1 year after its onset, which could be characterized by the long duration of exposure to stressful conditions. In our study, the observation of WB groups over time shows no differences in the percentage of individuals with different WB level from the first and second to the fourth waves. This is particularly interesting if we consider that it might have been possible to expect an increase in perceived WB during the fourth survey wave (i.e., May 2021), due to the fact that it was conducted when there was a much greater availability of vaccines, the pandemic was under more control by governments and healthcare services, and restrictions were gradually being phased out, with a partial return to “pre-pandemic” daily activities.. This result is different from that found by Fioravanti et al.^25^. Indeed, they found that WHO-5 score statistically significantly changed across waves in 2020 (WHO-5 scores: T0 March 2020 = 11.01 [SD = 5.05]; T1 May 2020 = 12.89 [SD = 4.95]; T2 November 2020 = 10.60 [SD = 4.72]) probably associated with social restrictions (i.e., the scores increased from March to May, and decreased from May to November). However, the mean WHO-5 scores found by the study of Fioravanti et al.^25^ were clinically almost stable across waves and quite similar to those found by our research study. It should be highlighted that the stability found in WHO-5 scores across the four waves stands in contrast to the wellbeing theory according to which human well-being is a multidimensional, subjective value concept that differs according to culture, gender, age, and time^26^. However, in our study WB was measured with a brief online questionnaire—specifically focused on psychological WB—which may have limited the chance of analyzing the impact of time on this dimension.

Worldwide, a large number of studies on the general population during the COVID-19 pandemic have mainly focused on the investigation of specific psychiatric symptoms or clinical states, such as anxiety, depression, insomnia, and post-traumatic stress^27–31^. Recent systematic reviews and meta-analyses on mental health problems seem to point to high depression rates (assessed in different ways) in the general population, ranging from 24^27^ to 33.7%^28^. In Italy, different studies on mental health and clinical symptoms have been conducted during the pandemic^11,25,32–37^, while very few focused on the WB status of citizens^11,25,36,38^. These studies found that COVID-19 significantly impacted WB of Italian citizens^36,38^ and that WB status was influenced by a variety of different individual and community factors, including social support, psychological flexibility, education levels, family functioning, inability to obtain all basic supplies, mindfulness, living with friends/roommates, gender, age, socioeconomic status, occupational status, coping efficacy, trust in the institutional response, and socioeconomic status^11,36^.

### Resilience and WB

Resilience plays a decisive role in the response of individuals under pressure and it can help them deal with it more effectively^39^. In this regard, the members of the European College of Neuropsychopharmacology (ECNP) highlighted the urgent need for a focus on resilience during the coronavirus pandemic^40^. Our study found that resilience was a key and stable factor able to discriminate WB levels in our general population sample across the 4 waves, demonstrating that greater resilience is associated with greater WB. This finding is in line with theory according to which WB is characterized by the ability to “respond constructively to social challenges and rapidly adjust behaviour in response to social cues and norms”^41^.

Similarly, an Italian study found negative correlations between different resilience factors and depression, anxiety, and stress during the COVID-19 pandemic^42^. Killgore et al.^43^ found that resilience levels were higher among those who tended to get outside more often, exercise more often, perceive more social support from family, friends, and significant others, sleep better and pray more often.

### Unhealthy behaviours and WB

The bidirectional relationship between unhealthy behaviours (i.e., smoking, unhealthy diet, drinking alcohol, sedentary, not seeking medical support) and WB has a long tradition and has been particularly emphasized in recent decades^44^. Our study found that *Increased Unhealthy Behaviours* were a leading factor to discriminate WB levels mainly in the first two waves (January and February 2021), demonstrating that people with "Poor WB” and “Depression” reported more *Increased Unhealthy Behaviours*. This finding is in line with theory according to which WB is characterized by the ability to “engage in sustained, constructive, self-controlled goal-directed activity within complex social environments, in ways that exercise skills, achieve valued or meaningful outcomes, and avoid chronic stress”^41^**.**

To date, few studies have been conducted in order to investigate the relationship between specific (un)healthy behaviour and WB or mental health. For example, studies on physical activity during the pandemic found that different levels of physical activity were associated with better mental health in different psychological domains (i.e., quality of life, anxiety, depression, WB, perceived stress)^6,45^. Furthermore, decreased vigorous physical activity during restrictions^24^, decreased nature-based and nature-neutral sports activities^46^, and not participating in any physical activity^47^ were associated with a decrease in WB or mental health. Similar results have been found for the association between mental health and dietary quality^45^, sleep quality^45^, and alcohol and cigarette consumption^48^ during the COVID-19 pandemic.

### COVID-19 perceived risk and WB

The way people perceive the risk of being infected can be significantly related to their mental health condition: in fact, our results show that people with "Good WB" have a lower perception of risk. Previous studies have found similar results, suggesting a strict relationship between *COVID-19 Perceived Risk* and several mental health domains in both general^49^ and vulnerable (i.e., COVID-19 patients, older people)^50,51^ population samples. In addition, the “PsyCorona Survey”, including 54,845 participants from 112 countries, found that risk perception at baseline was inversely associated with subsequent mental health outcomes^52^.

### Financial situation and WB

The COVID-19 pandemic and related restrictions had a significant economic impact on individuals and companies. *Financial Situation* became more relevant to discriminate WB levels in the last two waves, when the crisis was gradually reducing and reopening was taking place, and there was an accumulation of more people feeling the economic consequences of the pandemic lockdowns, showing that people with "Poor WB” and “Depression” reported more economic difficulties. Our findings are in line with previous studies that have suggested that individuals who, during the pandemic, had experienced economic losses^53^ or had a lower income^6^, had more mental health problems.

### Limitations

The limitations of this study include the lack of an assessment of individuals aged under 18 and over 70, and of specific personality traits and coping strategies that may be important in modulating WB levels. Including people older than 70 years would allow for generalization of our findings to the older Italian population, a specific vulnerable group that has been particularly affected by the pandemic and by the social restrictions and is at a generally higher risk for reduced WB (i.e., depression in particular). Unfortunately, it was not possible to involve this population for the online format of the survey. Further, using online panels limits the participation of other important population groups, including migrants, refugees, young people below 18 years, homeless people and other vulnerable groups, and so it cannot be claimed to represent their views, and the findings of the survey need to be interpreted in this specific context. It should also be noted that self-reported behaviours are known to differ from actual behaviour, not least due to the social desirability effect, and so the findings related to behaviour should be interpreted with this reliability limitation in mind. Moreover, an investigation of personality traits and coping strategies would help identify individual traits that may modulate the impact of the pandemic on subjective WB. Finally, the lack of data on participants’ mental health before the pandemic partially limits the interpretation of our results.

## Conclusions

Mental health problems have persisted at a high level even several months after the COVID-19 pandemic peaks in Italy. Given the large number of people experiencing poor WB, it is essential to ensure adequate mental health care and support during and after the COVID-19 pandemic for individuals at higher risk. To adequately address these needs, it is paramount to detect properly and robustly (here ensured by the application of machine learning techniques) the factors and variables directly involved in the WB status. Improving the level of individual resilience and the adoption of healthy behaviour may be beneficial for the prevention of psychological stress and may contribute to the improvement of mental health. More attention should be paid to individuals who have experienced financial loss during the pandemic, providing them with specific support. Finally, the critical lessons learned related to the negative impact of this pandemic on the mental health and well-being of broad population groups must be taken seriously and considered in any future health crisis response.

### Ethics approval and consent to participate

This study was approved by the Ethics Committee of the IRCCS San John of God Fatebenefratelli of Brescia (n° 286/2020), and all participants provided informed consent.

### Methodological statement

We confirm that all methods were carried out in accordance with relevant guidelines and regulations.

## Supplementary Information

Supplementary Information.

## Data Availability

Data and code to replicate the analysis are available here: [Data set]. Zenodo. http://doi.org/10.5281/zenodo.5040719. Data should be requested to the first author.
