# Supplementary material for: Psychological well-being during the COVID-19 pandemic in Italy assessed in a four-waves survey
Source: Sci Rep. 2022 Oct 26;12:17945. doi: 10.1038/s41598-022-22994-4 (PMC9606283; doi:10.1038/s41598-022-22994-4)
Supplement: Supplementary file 1 — Supplementary Information. [file 41598_2022_22994_MOESM1_ESM.docx]

**SUPPLEMENTARY MATERIALS AND METHODS**

**State of the pandemic in Italy during the study survey**

A brief description of the local pandemic situation may be useful to better place our study. Italy was the first European country with a major outbreak of COVID-19 starting at the end of February 2020, with the first wave lasting until May 2020 (with lockdown from early March to early May), and a second in October-December 2020, with estimated excess deaths of 31% and 35% respectively (Dorucci et al., 2021). The first vaccines were administered during the “European vaccination days” (27-29 December 2020), and vaccinations were rolled-out based on level of priority, starting from health workers, elderly and vulnerable people, then to essential workers and opening progressively to younger age groups until during July 2021 vaccination opened for anyone (see Epicentro).

The present study was conducted during the vaccine roll-out in Italy, from January to May 2021. In the period of the first wave (January 8-18, 2021) there were 339.2 new cases per 100,000 and 1,448 deaths out of 204,374 new cases (from 04/01/2021 to 17/01/2021). The situation improved during the second wave (February 8-19, 2021) with 134.3 new cases per 100,000 people and 823 deaths out of 163,538 new cases (from 01/02/2021 to 14/02/2021). During the third wave (March 23 - April 2, 2021) the situation worsened with 236.5 new cases per 100,000 people and 1,288 deaths out of 286,694 new cases (from 15/03/2021 to 28/03/2021). Finally, during the fourth wave (May 7-20, 2021), the situation had again improved, with 88 new cases per 100,000 people and 299 deaths out of 107,063 new cases (from 3/05/2021 to 16/05/2021) (data source: weekly reports of the National Institution for Health - ISS).

As for public health restrictions in place, following the waves of cases (October-December 2020) were applied based on a semaphore-like system (red, orange, yellow, white), based on the number of new cases and hospitalizations per region, updated weekly. These measures affected travelling between different regions; wearing masks was compulsory indoor and in many cases also outdoor; in schools the distance learning options were depending also on their grade; finally, for some months it was forbidden to consume food and drinks inside bars and restaurants, etc. As the number of new cases decreased during the four waves, these measures were lifted in most regions by the end of the study.

Finally, several financial aids to companies, traders, shop owners, etc. have been legislated during the different waves of the pandemic. While space reasons prevent us from a detailed account of these financial aids, there is a large agreement among politicians, trade unions, and journalists, that these financial aids have been fundamental to prevent a massive economic recession, with severe social and humane consequences on the general population.

**References**

Dorrucci, M., Minelli, G., Boros, S., Manno, V., Prati, S., Battaglini, M., ... & Bella, A. (2021). Excess mortality in Italy during the COVID-19 pandemic: assessing the differences between the first and the second wave, year 2020. Frontiers in public health, 927.

Epicentro (2021). National COVID-19 vaccination plan. <https://www.epicentro.iss.it/en/vaccines/covid-19-vaccination-plan>

Epicentro (2021). Epidemia COVID Aggiornamento 20 gennaio 2021. https://www.epicentro.iss.it/coronavirus/bollettino/Bollettino-sorveglianza-integrata-COVID-19_20-gennaio-2021.pdf

Epicentro (2021). Epidemia COVID-19 Aggiornamento nazionale 17 febbraio 2021. https://www.epicentro.iss.it/coronavirus/bollettino/Bollettino-sorveglianza-integrata-COVID-19_17-febbraio-2021.pdf

Epicentro (2021). Epidemia COVID-19 Aggiornamento nazionale 31 marzo 2021. https://www.epicentro.iss.it/coronavirus/bollettino/Bollettino-sorveglianza-integrata-COVID-19_31-marzo-2021.pdf

Epicentro (2021). Epidemia COVID-19 Aggiornamento nazionale 19 maggio 2021. https://www.epicentro.iss.it/coronavirus/bollettino/Bollettino-sorveglianza-integrata-COVID-19_19-maggio-2021.pdf

**WHO Questionnaire items**

We treated 7 points Likert scales in different ways: in some cases (i.e., variables related to Risk Perception, in Table 1S) we assigned 1-3 scores a positive meaning (*Yes)*, scores 5-7 a negative meaning (*No)* and grade 4 was classified as no response (*Don’t Know)*; in other cases instead (i.e., variables related to Trust & Use of sources of information, in Table 1S) we have considered Likert points as numbers. When we applied supervised machine learning approaches, we excluded the *Don’t Know* answers from the analyses (i.e., *Beliefs on vaccine efficacy*, in Table 1).

**Partial Least Square Discriminant Analysis (PLS-DA)**

Partial Least Square Discriminant Analysis is a classification technique (supervised machine learning techniques) that combines a Partial Least Square Regression (PLS-R) and Linear Discriminant Analysis (LDA); in particular, the PLS-DA is a PLS-R in which the dependent variable is categorical.

PLS-R is a method that at the same time allows dimension reduction and the fit of a regression model. It is an approach similar to Principal Component Analysis but, instead of a computation of a new variable (the principal component), a categorical response variable is used [1]. LDA is a method whose purpose is to find a linear combination that allows to discriminate two or more groups maximizing their separation.

Therefore, PLS-DA provides a dimension reduction in a discriminant application maximizing among-groups variability [2].

Formally, PLS-DA is based on a regression model between the data matrix $X$ and the vector of categories (the group variable) C.

The fundamental equations of PLS-DA are the following:

$$X=TP^{T}+E$$

$$C=TQ^{T}+f$$

where $T$ is the score matrix, $E$ and $f$ are the residuals and $P$ and $q$ are respectively the loadings of $X$ and of $C$ (for more details see [3]).

Once the model is built the class membership can be predicted through the equation

$\hat{C}=X^{T}\beta$,

where $\beta$ is the regression coefficient vector.

Therefore, a generic subject will be classified i.e. assigned to a category on the basis of the estimated value of c (and it will be assigned to the category with the nearest value).

The main advantage of PLS-DA, compared to linear discriminant analysis, is that it can provide also variables loadings (represented by a bar plot) that allow to identify not only the group a subject belongs to, but also which variables are more helpful to discriminate the subjects in the different classes [3].

**Classification Trees (CT)**

Classification trees are one of the most popular machine learning algorithms that belong to the family of decision trees, that can be used for both classification and regression purpose. CT are models in which the dependent variable (variable that has to be predicted) is categorical and the independent ones (covariates) can be categorical or quantitative.

Classification trees are directed graphs in which there is an initial node that branches to many. Each node represents an independent variable, each edge corresponds to a decision rule and each leaf represents an outcome (a value of the predicted variable). The top node contains all the sample that is consequently divided into different subsets. If the covariates are quantitative splits are created on the basis of some cut-offs on a scale; if the covariates are categorical, splits are based on the different categories [4].

After computing the entire tree (with all the independent variables) some techniques have to be used to reduce tree dimension and to improve the tree predictive power, reducing overfitting. Among these, one of the most used is p*runing* [5], a method that allows to remove the variables that do not contribute (are not significantly associated) to the final outcome, considering a penalty for the increase of parameters in the model.

Therefore, the final tree shows only the independent variables that are significant predictors of the dependent one (outcome) and, differently from the traditional regression models, those that are not predictors do not influence the final result.

The classification trees can be built by a recursive partitioning program using a two-stage procedure [6]:

1. The variable which best splits the data into groups (i.e. with the greatest association with the dependent variable) is found. The subjects are divided and this process is repeated separately to each subject subgroup recursively until the subgroups either reach a minimum size or until no improvement (in terms of predictive performance) can be made;
2. A cross-validation (*pruning*) will be performed to trim the full tree, since the full model is quite certainly too complex and overfitted.

For further detail see Ferrari et al. 2018 [7].

**s-References**

[1] Boulesteix AL. and Strimmer K. (2006) Partial least squares: a versatile tool for the analysis of high-dimensional genomic data. Brief Bioinform 8(1): 32-44.

[2] Barker M. and Rayens W. (2003) Partial least squares for discrimination*.* *J Chemom* 17: 166-173.

[3] Brereton RG. and Lloyd GR. (2014) Partial least squares discriminant analysis: taking the magic away. *J Chemom* 28: 213-225.

[4] Wilkinson L. (1992) *Tree Structured Data Analysis: AID, CHAID and CART*. Sawtooth/SYSTAT Joint Software Conference, Sun Valley, ID.

[5] Breiman L., Friedman J. H., Olshen R. A. and Stone C. J. (1984) *Classification and Regression Trees*. Wadsworth International Group, Belmont

[6] Therneau T.M, Atkinson E. Mayo Foundation (2018) *An introduction to Recursvie Partitioning Using the RPART Routines.* https://cran.r-project.org/web/packages/rpart/vignettes/longintro.pdf

[7] Ferrari C. et al. (2018) *Multivariate Statistical Techniques to Manage Multiple Data in Psychology. OAJ Behavioural Sci Psych 2018, 1(2): 180006.*

**Table 1****S.**

**Overall sample composition and selected characteristics according to WHO-5 scores (n=10,013).**

|  | **Good WB**  **[Group 2]**  **N=4,147**  **(41.4%)** | **Poor WB**  **[Group 1]**  **N=3,053**  **(30.5%)** | **Depression**  **[Group 0]**  **N=2,813**  **(28.1%)** | **Test** | **p-value** | **Bonferroni Post-Hoc** | **Effect size** |  |
| --- | --- | --- | --- | --- | --- | --- | --- | --- |
| **SOCIODEMOGRAPHIC** | | | | | | | | |
| *Age (mean, SD)* | 46.7 (12.7) | 44.7 (13.0) | 45.1 (12.9) | K-W | **<0.001** | 0/1<2 | .005 |  |
| *Sex*  Male  Female | 2,334 (56.3%)  1,813 (43.7%) | 1,486 (48.7%)  1,567 (51.3%) | 1,145 (40.7%)  1,668 (59.3%) | Χ^2^ | **<0.001** | £ $ & | .128 |  |
| *Educational Level (yrs)*  0-8 years  >8 years | 1,700 (41.0%)  2,447 (59.0%) | 1,156 (37.9%)  1,897 (62.1%) | 1,147 (44.3%)  1,566 (55.7%) | Χ^2^ | **<0.001** | £ $ & | .050 |  |
| *Occupational Status*  Yes  No | 2,272 (54.8%)  1,875 (45.2%) | 1,619 (53.0%)  1,434 (47.0%) | 1,364 (48.5%)  1,449 (51.5%) | Χ^2^ | **<0.001** | $ & | .052 |  |
| *Health workers*  Yes  No | 154 (6.8%)  2,118 (93.2%) | 137 (8.5%)  1,482 (91.5%) | 85 (6.2%)  1279 (93.8%) | Χ^2^ | **0.041** | / | .035 |  |
| *Italian Region*  North  Center  South & Islands | 1,970 (47.5%)  785 (18.9%)  1,392 (33.6%) | 1,401 (45.9%)  583 (19.1%)  1,069 (35.0%) | 1,268 (45.1%)  552 (19.6%)  993 (35.3%) | Χ^2^ | 0.336 | / | .021 |  |
| *Chronic Illness*  Yes  No  Don’t know | 781 (18.8%)  3,248 (78.3%)  118 (2.8%) | 643 (21.1%)  2,271 (74.4%)  139 (4.6%) | 735 (26.1%)  1,904 (67.7%)  174 (6.2%) | Χ^2^ | **<0.001** | $ & | .106 |  |
| *Cohabiting*  Alone  With other | 477 (11.5%)  3,670 (88.5%) | 293 (9.6%)  2,760 (90.4%) | 280 (10.0%)  2,533 (90.0%) | Χ^2^ | **0.018** | £ | .028 |  |
| *Financial Situation (over the past 3 months)*  Improved  Remains the same  Worse | 227 (5.5%)  2,809 (68.4%)  1,073 (26.1%) | 118 (3.9%)  1,797 (59.7%)  1,096 (36.4%) | 67 (2.4%)  1,417 (51.0%)  1,296 (46.4%) | Χ^2^ | **<0.001** | £ $ & | .181 |  |
| *Financial Concerns*  No  Yes  Don’t know | 755 (18.2%)  2,194 (52.9%)  1,198 (28.9%) | 294 (9.6%)  1,991 (65.2%)  768 (25.2%) | 252 (9.0%)  2,034 (72.3%)  527 (18.7%) | Χ^2^ | **<0.001** | £ $ & | .181 |  |
| **COVID-19 RISK PERCEPTION** | | | | | | | | |
| *Probability of getting infected with COVID-19*  Likely  Unlikely  Don’t know | 868 (20.9%)  1,139 (27.5%)  2,140 (51.6%) | 769 (25.2%)  651 (21.3%)  1,633 (53.5%) | 778 (27.7%)  575 (20.4%)  1,460 (51.9%) | Χ^2^ | **<0.001** | £ | .089 |  |
| *COVID-19 to me feels…*  Close to me  Far from me  Don’t know | 1,565 (37.7%)  1,248 (30.1%)  1,334 (32.2%) | 1,428 (46.8%)  655 (21.5%)  970 (31.8%) | 1,432 (50.9%)  564 (20.0%)  817 (29.0%) | Χ^2^ | **<0.001** | £ $ & | .128 |  |
| *COVID-19 to me feels…*  Fear-inducing  Not fear-inducing  Don’t know | 2,605 (62.8%)  734 (17.7%)  808 (19.5%) | 2,146 (70.3%)  380 (12.4%)  527 (17.3%) | 2,121 (75.4%)  302 (10.7%)  390 (13.9%) | Χ^2^ | **<0.001** | £ $ & | .117 |  |
| **COVID-19 EXPERIENCE** | | | | | | |  |  |
| *COVID-19 personal infection*  Yes  No  Don’t know | 295 (7.1%)  3,549 (85.6%)  303 (7.3%) | 235 (7.7%)  2,543 (83.3%)  275 (9.0%) | 223 (7.9%)  2,316 (82.3%)  274 (9.7%) | Χ^2^ | **0.002** | £ | .041 |  |
| *Connections affected by COVID-19*  Yes, but not died  Yes, and died  No | 1,307 (31.5%)  1,621 (39.1%)  1,219 (29.4%) | 1,013 (33.2%)  1,306 (42.8%)  734 (24.0%) | 899 (32.0%)  1,159 (41.2%)  755 (26.8%) | Χ^2^ | **<0.001** | £ | .051 |  |
| **TRUST IN INFORMATIONAL SOURCES** | | | | | | |  |  |
| *Non-institutional* | 3.5 (1.2) | 3.5 (1.1) | 3.3 (1.2) | K-W | **<0.001** | 0<1/2 | .004 |  |
| *Institutional* | 4.9 (1.3) | 4.8 (1.1) | 4.7 (1.2) | K-W | 0.122 | **0<1<2** | .003 |  |
| **FREQUENCY OF USE OF INFORMATIONAL SOURCES** | | | | | | |  |  |
| *Non-institutional* | 3.2 (1.2) | 3.3 (1.1) | 3.1 (1.2) | K-W | **<0.001** | 0<1/2 | .003 |  |
| *Institutional* | 3.7 (1.4) | 3.7 (1.2) | 3.5 (1.4) | K-W | **<0.001** | 0<1/2 | .003 |  |
| **TRUST IN HEALTHCARE INSTITUTIONS** | | | | | | |  |  |
| *Trust in Healthcare Institutions (Family doctors, Hospitals, Ministry of Health, Institute of Public Health)* | 4.9 (1.1) | 4.8 (1.0) | 4.6 (1.2) | K-W | **<0.001** | **0<1<2** | .011 |  |
| **OTHER AREAS** | | | | | | | | |
| *Resilience* | 4.5 (1.1) | 3.9 (1.0) | 3.4 (1.1) | K-W | **<0.001** | **0<1<2** | **.150** |  |
| *Increased Unhealthy Behaviours*  No  Yes (TWO items) | 2,772 (66.8%)  1,375 (33.2%) | 1,667 (54.6%)  1,386 (45.4%) | 1,358 (48.3%)  1,455 (51.7%) | Χ^2^ | **<0.001** | £ $ & | .160 |  |
| *Beliefs on vaccine efficacy in reducing spread of COVID-19*  *Yes*  *No*  *Don’t know* | 3,192 (77.0%)  402 (9.7%)  553 (13.3%) | 2,355 (77.1%)  216 (7.1%)  482 (15.8%) | 2,184 (77.6%)  243 (8.6%)  386 (13.7%) | Χ^2^ | **<0.001** | £ | .047 |  |

Table 1S. Between groups differences for WB status.

*Percentages are shown by rows

£: Good vs Poor; $ Good vs dep; &: poor vs Dep;

**Table 2S.**

**Variable rankings, based on the performance in discriminating the well-being groups, obtained by the three supervised machine learning approaches at different waves.**

| **WAVE 1-2** | **Logistic regression** | | | **PLS-DA** | | **Classification tree** | |
| --- | --- | --- | --- | --- | --- | --- | --- |
|  | 1. Resilience 2. Increased Unhealthy- Behaviours 3. Financial Situation | | | 1. Resilience 2. Increased Unhealthy- Behaviours 3. COVID-19 Perceived Risk | | 1. Resilience 2. Trust in Healthcare Institutions 3. COVID-19 Perceived Risk | |
| **WAVE 3** | **Logistic regression** | | | **PLS-DA** | | **Classification tree** | |
|  | 1. Resilience 2. Financial Situation 3. Increased Unhealthy- Behaviours | | | 1. Resilience 2. Financial Situation 3. Increased Unhealthy- Behaviours | | 1. Resilience 2. Financial Situation 3. Trust in Healthcare Institutions | |
| **WAVE 4** | **Logistic regression** | | | **PLS-DA** | | **Classification tree** | |
|  | 1. Resilience 2. Financial Situation 3. COVID-19 Perceived Risk | | | 1. Resilience 2. Financial Situation 3. COVID-19 Perceived Risk | | 1. Resilience 2. Financial Situation 3. Age | |
|  | | **Resilience** | **COVID-19 Perceived Risk** | | **Increased Unhealthy- behaviours** | | **Financial Situation** |
| **WAVE 1-2** | | 1^st^ | 3^rd^ | | 2^nd^ | | 4^th^ |
| **WAVE 3** | | 1^st^ | Not important | | 3^rd^ | | 2^nd^ |
| **WAVE 4** | | 1^st^ | 3^rd^ | | Not important | | 2^nd^ |

**Table 3S.**

**Factor analysis carried out for obtaining COVID-19 Perceived Risk and Trust in Health Institution new variables.**

|  | **COVID-19 Perceived Risk** | **Trust in Healthcare Institutions** |
| --- | --- | --- |
| **N. of Items** | 5 | 4 |
| ***Cronbach's* α** | 0.748 | 0.880 |
| **Item 1** | Probability | Family doctor |
| **Item 2** | Vulnerability | Local Health  Unit |
| **Item 3** | Severity | Health Ministry |
| **Item 4** | Affect-Frightening | National Health  Institute (ISS) |
| **Item 5** | Affect-Closeness |  |
| **Load. Item 1** | 0.491 | 0.464 |
| **Load. Item 2** | 0.537 | 0.581 |
| **Load. Item 3** | 0.464 | 0.901 |
| **Load. Item 4** | 0.557 | 0.933 |
| **Load. Item 5** | 0.546 |  |
| **Percentage of**  **Explained variability** | 0.42 | 0.65 |
| **Mean (SD)** | 0 (1) | 0 (1) |

*Number of items, internal consistency (Cronbach’s α), items name and their estimated loadings, total deviance explained by the loadings and proportion of variance explained by EFA for each dimension.*

**Table 4S.**

**Factor analysis details and differences in items’ loadings among waves.**

| **Index** | **Wave** | **N** | **N items** | **Alpha** | **Var. Exp** | **Load1** | **Load2** | **Load3** | **Load4** | **Load5** |
| --- | --- | --- | --- | --- | --- | --- | --- | --- | --- | --- |
| COVID-19 Perceived Risk | 1-2 | 5006 | 5 | 0.633 | 41% | 0.49 | 0.54 | 0.46 | 0.56 | 0.55 |
| COVID-19 Perceived Risk | 3 | 2507 | 5 | 0.748 | 42% | 0.49 | 0.53 | 0.48 | 0.58 | 0.52 |
| COVID-19 Perceived Risk | 4 | 2500 | 5 | 0.720 | 39% | 0.46 | 0.43 | 0.46 | 0.60 | 0.48 |
| Trust in Healthcare Institutions | 1-2 | 5006 | 4 | 0.880 | 65% | 0.46 | 0.58 | 0.90 | 0.93 | *NA* |
| Trust in Healthcare Institutions | 3 | 2507 | 4 | 0.888 | 67% | 0.50 | 0.61 | 0.92 | 0.93 | *NA* |
| Trust in Healthcare Institutions | 4 | 2500 | 4 | 0.884 | 66% | 0.51 | 0.67 | 0.86 | 0.90 | *NA* |

*Factor, wave, number of items, internal consistency (Cronbach’s α), proportion of variance explained and items estimated loadings.*

**Table 5S.**

**Studies conducted administering the WHO-5 to general population samples during the pandemic.**

| **AUTHOR YEAR** | **COUNTRY** | **SAMPLE**  **(N)** | **DESIGN^1^** | **SURVEY TIMEFRAME** | **AGE GROUP** | **WHO-5 SCORE RANGE** | **WHO-5 CUTOFF FOR GROUP CATEGORI-ZATION** | **GOOD WB**  **(%)** | **POOR WB**  **(%)** | | **THRESHOLD FOR DEPRESSION (%)** |
| --- | --- | --- | --- | --- | --- | --- | --- | --- | --- | --- | --- |
| Andersen et al. 2021 | Denmark | 2,836* | CS | March - April 2020;  July 2020 | ≥18 | 0-100 | <50= Depression  >50= WB | 80 | 20 | | |
| Bhowmick et al. 2021 | India | 355 | SCS | April – May 2020 | ≥18 | 0-25 | <12= low WB | NA | 37.74 | | |
| Büssing et al. 2020 | Germany | 1,277 | SCS | June 2020 | 15-92 | 0-25 | <13= low WB  13-18= moderate WB  >18= WB. | 30.8 | 39.5 | | 29.7 |
| Büssing et al. 2021 | Germany | 2,573 | SCS | June - November, 2020 | NA | 0-25 | <13= low WB  13-18= moderate WB  >18= WB | 29.7 | 37.8 | | 32.5 |
| Dale et al. 2021 | Austria | 1,505 | SCS | December 2020 - January 2021 | ≥18 | 0-25 | NA | Mean 14.3 (SD 5.8) | | | |
| Dawel et al. 2020 | Australia | 1,296 | SCS | March 2020 | ≥18 | 0-25 | NA | Mean 11.9 (SD 5.9) | | | |
| Every-Palmer et al. 2020 | New Zealand | 2,010 | SCS | April 2020 | ≥18 | 0-25 | <13= low WB  13-21=moderate WB  >21= excellent WB | 8.7 | 56.1 | 38.2 | |
| Eric et al. 2020 | Nigeria | 1,800 | SCS | NA | NA | 0-100 | ≤28= low WB  ≤50= moderate WB  >50= WB | 48.7 | 23.3 | 27.0 | |
| Faruk et al. 2021 | Bangladesh | 422 | SCS | January – April, 2021 | 16-90 | NA | NA | NA | 50.9 (“low WB”) | | |
| Fioravanti et al. 2022 | Italy | 1,258 | MWS | May 2020 – January 2021 | 18-70 | 0-25 | NA | T0: Mean 11.01 (SD 5.05)  T1: Mean 12.89 (SD 4.95)  T2: Mean 10.60 (SD 4.72) | | | |
| Gao et al. 2020 | China | 4,872 | SCS | January – February 2020 | 18-85 | 0-25 | <13= Depression | NA | 48.3 | | |
| Hoang et al. 2021 | Vietnam | 1,922 | SCS | April 2020 | 18-76 | 0-25 | <13= Poor WB  ≥ 13 = WB | 83.9 | 16.1 | | |
| Jung et al. 2020 | Germany | 3,545 | SCS | April 2020 | NA | NA | NA | Mean 51.4 (SD 23.9) for women  Mean 47.5 (SD 22.5) for men | | | |
| Khan et al. 2021 | Pakistan | 1,756 | SCS | April – May, 2020 | NA | 0-100 | <50= Poor WB  >50= WB | 58.8 | 41.2 | | |
| Pieh, Budimir, Probst, 2020 | Austria | 1,005 | SCS | March-April 2020 | ≥18 | 0-25 | NA | Mean 15.0 (SD 5.4) | | | |
| Pieh, Budimir, Humer, Probst, 2021 | Austria | 1,005*  437** | CS | April – September, 2020 | ≥18 | 0-25 | NA | 1^st^ assessment: mean 15.4 (SD 5.6)  2^nd^ assessment: mean 15.9 (SD 5.8) | | | |
| Simon et al. 2021 | Austria | 560 | SCS | March - April 2020 | 18-79 | 0-25 | <13= low WB | NA | NA | | |
| Wilke et al. 2021 | 14 countries | 14,975 | SCS | April – May 2020 | ≥18 | 0-25 | <50= Depression  >50= WB | 85.8***  54.8**** | 14.2***  45.2**** | | |

Legend:

WB=Well-Being

NA=Not Available

Design: SCS: Single Cross-Sectional study; CS: Prospective Cohort study; MWS: Multiple-wave survey.

*Sample at T0

**Sample at T0 and T1

***Before lockdown

****During lockdown

**Table 6S.**

**Table of instruments.**

| **Instrument** | **Construct** | **Objective** |
| --- | --- | --- |
| Sociodemographic form | Sociodemographic data (i.e., age, sex education level, occupational status, Italian region, chronic illness, cohabiting, and financial situation). | To investigate how the three groups of well-being (i.e. good WB, poor WB, depression) differentiate from each other in terms of sociodemographic characteristics. |
| Financial Concerns | Ad-hoc developed question on financial concerns (i.e. how much the individual is concerned about their own future financial situation) | To investigate how the three groups of well-being (i.e. good WB, poor WB, depression) differentiate from each other in terms of financial concerns. |
| COVID-19 Experience | Ad-hoc developed question on the personal direct and indirect experience with COVID-19 (i.e. having been infected or knowing someone infected). | To investigate how the three groups of well-being (i.e. good WB, poor WB, depression) differentiate from each other in terms of COVID-19 personal experience. |
| COVID-19 Perceived Risk | Ad-hoc developed questions on the perception of the Probability, Vulnerability, and Severity of getting related-affect (i.e. Affect-Frightening and Affect-Closeness). | To investigate how the three groups of well-being (i.e. good WB, poor WB, depression) differentiate from each other in terms of COVID-19 perceived risk. |
| Beliefs on vaccine efficacy | Ad-hoc developed question on beliefs about vaccine efficacy in reducing the spread of COVID-19. | To investigate how the three groups of well-being (i.e. good WB, poor WB, depression) differentiate from each other in terms of beliefs about vaccine efficacy. |
| Trust in Healthcare Institutions | Ad-hoc developed questions on trust in healthcare institutions (i.e., family doctors, hospitals, Ministry of Health, National Health Institute). | To investigate how the three groups of well-being (i.e. good WB, poor WB, depression) differentiate from each other in terms of trust in healthcare institutions. |
| Increased Unhealthy Behaviours | Ad-hoc developed questions on changes in unhealthy behaviours (i.e., physical activity, diet, smoking, drinking alcohol, medical seeking) during the previous two weeks | To investigate how the three groups of well-being (i.e. good WB, poor WB, depression) differentiate from each other in terms of frequency of increased unhealthy behaviours. |
| Brief Resilience Scale | Resilience | To investigate how the three groups of well-being (i.e. good WB, poor WB, depression) differentiate from each other in terms of Resilience. |
| WHO 5-item Well-Being Scale (WHO-5) | Well-being status in the previous two weeks | To investigate the percentages of WB across 4 waves in Italy.  To identify the variables that discriminate different levels of WB. |

**Figure 1S.**

**Boxplot of all WHO-5 scores per each wave.**


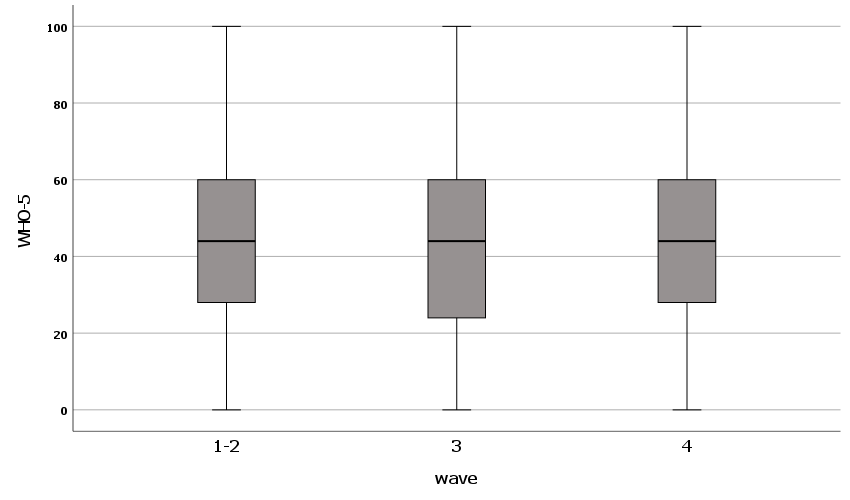


| **WHO-5 Percentiles across waves:** | **Waves 1-2** | **Wave 3** | **Wave 4** |
| --- | --- | --- | --- |
| **25^th^** | **28** | **24** | **28** |
| **50^th^** | **44** | **44** | **44** |
| **75^th^** | **60** | **60** | **60** |
